# COVID-19 in Pregnancy: Influence of Body Weight and Nutritional Status on Maternal and Pregnancy Outcomes—A Review of Literature and Meta-Analysis

**DOI:** 10.3390/nu15041052

**Published:** 2023-02-20

**Authors:** Rossella Attini, Maria Elena Laudani, Elisabetta Versino, Alessio Massaro, Arianna Pagano, Francesca Petey, Alberto Revelli, Bianca Masturzo

**Affiliations:** 1 Department of Obstetrics and Gynecology SC2U, Città della Salute e della Scienza, Sant’Anna Hospital, 10126 Turin, Italy; 2 Department of Clinical and Biological Sciences, University of Turin, 10100 Turin, Italy; 3 Centre for Biostatistics, Epidemiology and Public Health (C-BEPH), 10100 Turin, Italy; 4 Department of Obstetrics and Gynecology SC1U, Città della Salute e della Scienza, Sant’Anna Hospital, 10126 Turin, Italy; 5 Department of Maternal, Neonatal and Infant Medicine, University Hospital “Degli Infermi”, 13875 Ponderano, Italy

**Keywords:** COVID-19, pregnancy, nutritional status, BMI, severe COVID-19, micronutrients, maternal death, hospital admission, obesity, vitamin D

## Abstract

In the last two and a half years, COVID-19 has been one of the most challenging public health issues worldwide. Based on the available evidence, pregnant women do not appear to be more susceptible to infection than the general population but having COVID-19 during pregnancy may increase the risk of major complications for both the mother and the fetus. The aim of this study is to identify the correlation between BMI and nutritional status and the likelihood of contracting COVID-19 infection in pregnancy, its severity, and maternal pregnancy outcomes. We carry out a systematic literature search and a meta-analysis using three databases following the guidelines of the Cochrane Collaboration. We include 45 studies about COVID-19-positive pregnant women. Compared with normal-weight pregnant women with COVID-19, obesity is associated with a more severe infection (OR = 2.32 [1.65–3.25]), increased maternal death (OR = 2.84 [2.01–4.02]), and a higher rate of hospital admission (OR = 2.11 [1.37–3.26]). Obesity may be associated with adverse maternal and pregnancy outcomes by increasing symptom severity and, consequently, hospital and Intensive Care Unit (ICU) admission, and, finally, death rates. For micronutrients, the results are less definite, even if there seems to be a lower level of micronutrients, in particular Vitamin D, in COVID-19-positive pregnant women.

## 1. Introduction

In the last few years, the disease caused by SARS-CoV-2 (COVID-19) has presented one of the most challenging public health issues worldwide. The first case of COVID-19 infection was reported in Wuhan, Hubei Province, China, in December 2019, and the infectious disease was classified as a global pandemic by the World Health Organization (WHO) in March 2020 [1]. Thereafter, a high number of new cases and deaths due to COVID-19 were rapidly reported worldwide, affecting the general population as well as pregnant women.

According to the most recent Royal College of Obstetricians and Gynecologists (RCOG) Guidelines on COVID-19 infection in pregnancy, updated in January 2022, pregnant women appear as susceptible to contracting the infection as the general population, and most importantly, more than two-thirds of these women have no severe symptoms; when symptomatic, they generally complain of mild fever and cough [2]. However, a recent systematic review showed that COVID-19 infection in pregnancy is significantly associated with an increased incidence of unfavorable outcomes, such as pre-eclampsia, gestational diabetes, stillbirth, preterm birth, and low birthweight [3]. There is growing evidence that the rather small proportion of pregnant women who display fully symptomatic disease may be at a higher risk of severe complications than their non-pregnant counterparts, particularly in the third trimester of pregnancy, although the overall risk of death remains quite low [2].

Indeed, pregnancy involves unique physiological changes that include the partial suppression of the immune system, on the one hand allowing the body to tolerate the antigenically diverse fetus, while on the other increasing sensitivity to infections. It is well known that most immune cells possess receptors for steroid hormones: the progressively increasing estrogen and progesterone secretion of placental origin modulates the immune response, leading to transient immunosuppression. As a consequence, the mother and fetus become more susceptible to all kinds of infections [4]. Even if the placental barrier effectively prevents viruses from reaching the fetus and causing direct damage, the response of the mother’s immune system itself may negatively affect fetal development [5], thereby increasing the risk of adverse events (miscarriage, pre-eclampsia/eclampsia, intrauterine growth restriction, premature delivery, etc.) [6].

The link between nutrition in pregnancy and COVID-19 infection is suggested by the evidence that the immune response is significantly weakened by an inadequate or imbalanced intake of micronutrients, such as oligoelements and vitamins. The lack or shortage of vitamins and minerals throughout pregnancy may increase the risk of contracting a viral infection and worsening its severity, finally resulting in a higher incidence of pregnancy-related complications [7,8]. Further, a typical nutrition-linked disorder, obesity, has been reported to be a major risk factor for severe COVID-19 disease in pregnancy [9].

Moreover, since implantation and during pregnancy, there are changes in the physiological inflammatory reaction with an increase of oxidative stress that normally is well balanced by the action of reacting oxygen species (ROS) [10,11]. It is reasonable to think that the inflammation mechanism involved in COVID-19 infection, which could be responsible for adverse outcomes [12], may play a synergic role with adiposity-related inflammation in impairing the fine balance of pregnancy.

Taken together, these data suggest that normal body weight and adequate nutrition could be effective in empowering the maternal immune system, thus, better protecting women against all infections, including COVID-19.

In this review and meta-analysis, we analyze how women’s nutritional status (pre-pregnancy overweight/obesity and nutritional deficiencies) may influence the likelihood of contracting COVID-19 in pregnancy, disease severity, and maternal and pregnancy outcomes.

## 2. Methods

The search strategy followed the guidelines of the Cochrane Collaboration and was deliberately broad in order to increase sensitivity and to include all published articles about COVID-19 infection in pregnancy that reported pre-pregnancy body weight, maternal outcome, fetal outcome, or both. Database-specific searches were applied to PubMed, Embase, and Cochrane Central Register of Controlled Trials, analyzing the available literature published between March 2020 and August 2022.

Search terms were used as free terms. Terms referring to pregnancy, COVID-19, Body Mass Index (BMI), and nutrition were combined with ‘OR’; terms referring to all three were combined with ‘AND’. The following search string was used: (pregnancy OR gestation OR preeclampsia OR stillbirth OR gestational) AND (COVID-19 OR SARS-CoV-2) and (nutrient OR nutrition OR iron OR vitamin OR micronutrients OR minerals OR malnutrition OR diet OR oligoelements OR nutritional OR BMI OR overweight OR obesity). An additional manual search was carried out on the references of the reviewed studies to allow us to identify any additional papers that might have been missed in previous searches. No limits were placed on the search, which was performed in duplicate (by working independently and matching the results).

Abstracts and titles were screened in duplicate by two independent researchers who compared and matched their results and then agreed upon the final selection of the articles. Selected articles were divided into two major categories: those reporting COVID-19 incidence and severity in overweight/obese women and those reporting micronutrient circulating levels, micronutrient intake, and COVID-19 disease.

The following data were extracted: (a) baseline data: title, author, journal, year, country, main objective, study period (as stated in the paper), multicenter or single-center, type of study, number of cases, control group; (b) maternal infection in obese women: death, COVID-19 severity (according to the study definition); (c) micronutrients: any kind of micronutrient deficiency in the mother, nutritional supplementation(s); (d) pregnancy, maternal, fetal, and neonatal outcomes: hypertension, pre-eclampsia, proteinuria, gestational age at delivery, birthweight, preterm delivery, malformations, stillbirth, small for gestational age, admission to neonatal Intensive Care Unit (NICU), other neonatal complications (whenever reported), neonatal death.

In our analysis, we included all articles reporting at least one maternal and/or fetal outcome.

Two reviewers assessed the studies’ quality through the Newcastle–Ottawa Scale independently (NOS) [13]. Controversies were resolved by a third reviewer.

We used the threshold for converting the NOS to AHRQ standards (good, fair, and poor).

Every time it was possible, we performed a meta-analysis producing a pooled estimate of the effect size; in the case of studies’ data not being suitable for meta-analysis, results were presented in a narrative fashion.

Meta-analysis was performed using RevMan 5.4 software [14]

Data are shown as Mantel–Haenszel (M-H) odds ratios (95% CIs) in the case of dichotomous outcomes and as inverse variance (IV) standardized mean differences (95% CIs) in the case of continuous outcomes.

We evaluated heterogeneity with the I^2^ statistic. If the I^2^ value was 40% or greater [14], we considered heterogeneity to be present, and thus, we used a random-effects model to pool the data. We performed a sensitivity analysis that used a fixed-effects model for outcomes from studies with small numbers of patients (<100 per arm).

We used funnel plots to assess publication bias.

### Ethics Approval

Ethics approval was not sought for this systematic review because the data were publicly available.

## 3. Results

**Figure 1:**
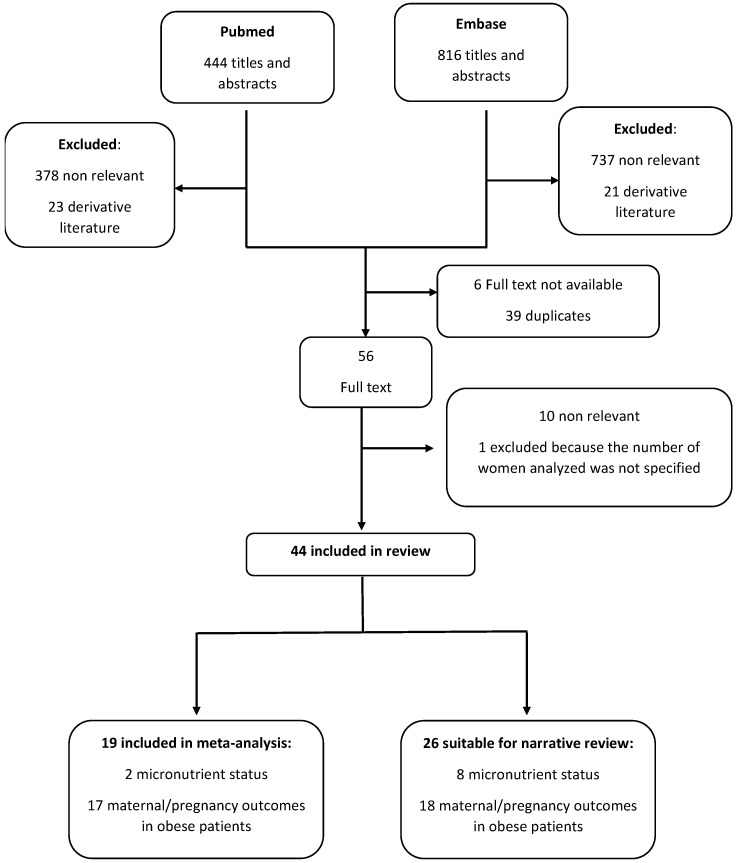
Flow chart for the selection of studies.

A total of 1260 pertinent titles were retrieved and screened; 56 articles were selected to be considered in full; and 45 more were identified from the reference lists. Finally, the selection process resulted in 35 articles reporting maternal and/or fetal outcomes in overweight/obese women and 10 articles reporting micronutrient deficiency/supplementation in pregnancy (Figure 1). The main characteristics of the studies not suitable for meta-analysis included in the review are reported in Table 1, Table 2, Table 3, Table 4 and Table 5.

The geographic origin of the 35 overweight/obesity studies was the following: 12 from North America, 7 from Central–South America, 13 from Europe, 1 from Asia, 1 from Africa, and 1 was multinational. Six of the ten studies on micronutrients came from Turkey and four from Europe. Most studies were carried out at single centers. The number of women included in the studies regarding overweight/obesity ranged from 5 to 3889, while in studies on micronutrients, it ranged from 15 to 448.

Most studies reported maternal outcomes, while none of the studies analyzed both maternal and fetal outcomes.

### 3.1. Meta-Analysis

Disease severity (critical/severe vs. mild)

**Figure 2:**
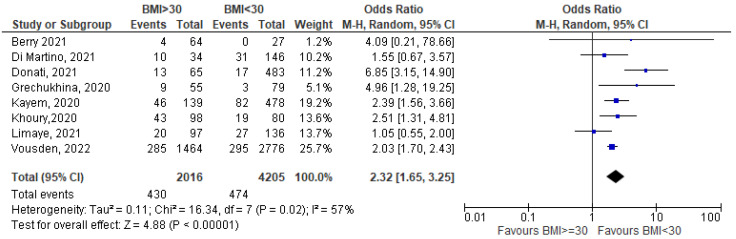
BMI >= 30 vs. BMI < 30—disease severity (critical/severe vs. mild) [15,16,17,18,19,20,21,22].

Pooled data show a statistically significant excess of risk of having critical/severe disease for obese (BMI >= 30) pregnant women (OR = 2.32 [1.65–3.25]; I^2^ = 57%; based on eight studies) [15,16,17,18,19,20,21,22] (Figure 2).

Maternal death due to COVID-19-related causes

**Figure 3:**
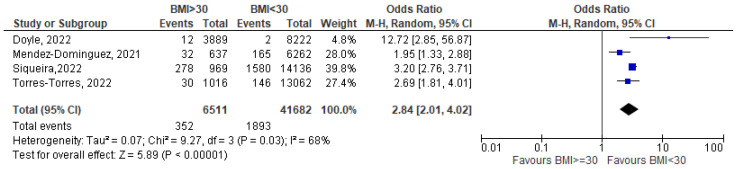
BMI >= 30 vs. BMI < 30—maternal death due to COVID-19-related causes) [23,24,25,26].

Pooled data show a statistically significant excess of risk of maternal death for obese pregnant women (OR = 2.84 [2.01–4.02]; I^2^ = 68%; based on four studies) [23,24,25,26] (Figure 3).

Hospital admissions for COVID-19-related causes

**Figure 4:**
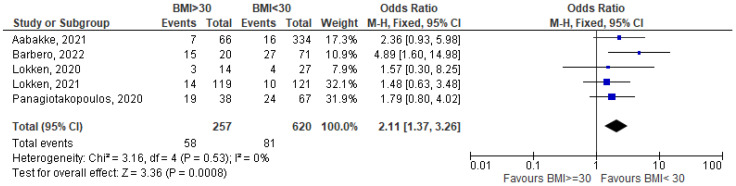
BMI >= 30 vs. BMI < 30—hospital admissions for COVID-19-related causes [27,28,29,30,31].

Pooled data show a statistically significant excess of risk of hospital admission for obese pregnant women (OR = 2.11 [1.37–3.26]; I^2^ =0%; based on five studies) [27,28,29,30,31] (Figure 4).

Vitamin D serum levels

**Figure 5:**
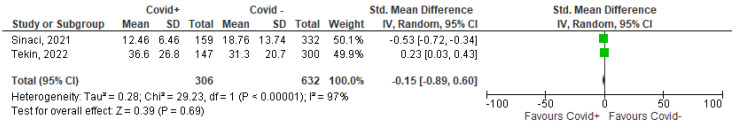
COVID-19-positive pregnant women vs. COVID-19-negative pregnant women—vitamin D serum levels [32,33].

Pooled data show that COVID-19-positive pregnant women have lower vitamin D serum levels when compared with COVID-19-negative pregnant women, but the result is not statistically significant (SMD = −0.15 [−0.89–0.60]; I^2^ = 97%; based on two studies) [32,33] (Figure 5).

Sensitivity analysis for all the outcomes goes in the same direction as the main analysis (Appendix A).

The funnel plot for hospital admissions suggests the absence of publication bias (Appendix B).

### 3.2. Narrative Review

**Incidence and severity of COVID-19 infection in overweight/obese pregnant women**

Thirty-five articles analyzed the relationship between high BMI (overweight or obesity) and COVID-19 infection incidence and severity. Unfortunately, none of the selected articles were specifically focused on COVID-19 in pregnant women with high BMI; in fact, data on overweight/obese women were extrapolated from large studies on COVID-19 in pregnancy, some of which included obese subjects.

Moreover, the definition of COVID-19 disease severity was highly heterogeneous: some articles simply divided patients into asymptomatic or symptomatic, without specifying the severity of symptoms; in others, the disease was classified as mild, moderate, or critical; and in three articles [25,34,35], the definition of severity was not reported.

**Pregnancy outcomes**

Only four of the reviewed studies reported pregnancy outcomes.

Three studies reported the pregnancy outcome of obese patients contracting COVID-19 infection during gestation. Vimercati showed that even if BMI was not associated with COVID-19 severity, a higher BMI was significantly associated with preterm birth [36]. Lokken reported the case of one woman with class III obesity (in a cohort of 15 patients), in whom preterm birth was induced to face the progressive worsening of respiratory function [30]. Stenton reported that COVID-19 infection in pregnant obese women was associated with a higher risk of fetal loss vs. pregnant non-obese women (67% vs. 41%) [37].

One study reported pregnancy outcomes in relation to micronutrient deficiency: Citu et al. found a correlation between a lack of magnesium and preterm labor (*p* = 0.038) [38].

**Micronutrient status**

We found 10 articles in which micronutrients were considered in relation to COVID-19 disease in pregnant patients. Different aspects were studied: circulating levels of zinc [39,40,41], copper [39,40,41], vitamin D [32,33,40,42], selenium [43], vitamin K1 [41], vitamin E, and Afamin (vitamin E-binding protein) [44]; deficiency of vitamin D [33,40,42,45] or vitamin B12 [40]; and supplementation of vitamin D [40,42] or magnesium [38]. The number of women with COVID-19 infection in each study was relatively small (15–448). The three largest studies analyzed magnesium supplementation (448 women) [38] or vitamin D circulating levels (491 and 347 women) [32,33]. The former reported a significant association between the lack of magnesium and preterm labor (*p* = 0.038) [38].

Overall, the heterogeneous definitions, design, and results of the published studies precluded the possibility of pooling data and, thus, identifying a clear relationship between micronutrients and the risk of contracting any degree of COVID-19 severity—from mild to severe. The available studies suggest only a slight relationship between low circulating levels of zinc, copper, vitamin B12, vitamin E, and selenium and the likelihood of contracting a severe form of COVID-19 infection.

**Table 1:** Association of overweight/obesity with COVID-19 illness severity.

	First Author	Country	Objective(s)	Women with COVID-19	Definition of Disease Severity	Disease Severity Status	Obese/Overweight Women	Mortality in Obese Women	Obstetric Outcome in Obese Women	Findings	
										Results	Comments as Reported in the Paper
1	Babic 2022 [46]	Saudi Arabia	To analyze the relationship between BMI and symptoms	209	Symptomatic: fever, cough, dyspnea, headache, sore throat, diarrhea, anosmia and/or ageusia, nausea, vomit, dizziness, rhinorrhea, myalgia	62 symptomatic	53 overweight 112 obese			BMI and symptoms *P* = 0.973 for overweight *P* = 0.985 for obesity	Overweight or obese women had no higher incidence of symptoms
2	Donati 2022 [47]	Italy	To describe COVID-19 infection among pregnant women and the impact of virus variants on the severity of maternal and perinatal outcomes	3306	COVID-19 pneumonia	424 COVID-19 pneumonia	427 obese	Not reported	Not reported	Pneumonia and obesity OR 1.72 (1.29–2.27)	Obesity was associated with a higher occurrence of pneumonia due to COVID-19
3	Galang 2021 [48]	USA	To determine risk factors for illness severity among pregnant women with COVID-19	7950	Critical: mechanical ventilation/intubation, ECMO, ICU admission, ARDS, respiratory failure, septic shock, MOF, COVID-19 listed as cause of death Moderate-to-severe: not meeting criteria for critical; presence of dyspnea/shortness of breath and at least one among fever or cough Mild: symptomatic not meeting criteria of critical or moderate-to-severe	512 with moderate-to severe or critical illness	1974 obese		Not reported	Moderate to severe or critical disease and death RR 1.36 (1.23–1.51)	Pre-pregnancy obesity associated with moderate-to-severe or critical COVID-19 disease
4	Eskenazi 2022 [49]	Multinational	To determine whether diabetes mellitus and high BMI are risk factors for COVID-19 in pregnancy	672	Symptomatic: any among chest pain, diarrhea or vomiting, limb or joint pain, sore throat, flu-like symptoms, runny nose, breathlessness, headache, tiredness or lethargy, loss of smell, fever, cough	400 symptomatic	328 with BMI > 28 208/328 symptomatic	Not reported	Not reported	BMI >28 and symptomatic disease: RR 1.6 (1.01–1.11)	Women overweight or obese more likely to develop symptomatic COVID-19
5	Menezes 2020 [50]	Brazil	To evaluate clinical and social risk factors associated with negative outcomes of COVID-19 disease in pregnancy	2475	Adverse composite outcome: critical disease leading to death or admission to ICU or mechanical ventilation	590 adverse outcome	116 obese; 48/116 with adverse composite outcomes	Included in adverse composite outcome	Not reported	Obesity and adverse composite outcome: OR 2.124 (1.381–3.268) *p* = 0.0006	Obesity associated with increased risk of adverse composite outcome
6	Overtoom 2022 [51]	Netherlands	To describe characteristics, risk factors and maternal, obstetric and neonatal outcomes of pregnant women with COVID-19	376	Need for hospitalization	74 hospitalized for COVID-19 6/62 admitted to ICU	100/376 overweight, 67/376 obese 25/74 hospitalized were overweight, 19/74 hospitalized were obese	Not reported	Not reported	Severe illness and BMI > 28 OR 1.86 (1.51–3.20)	Having BMI >28 is a risk factor in severe COVID-19 (need for hospitalization)
7	Péju 2022 [52]	France	To assess the ventilatory management of pregnant women with COVID-19 admitted to the ICU and report on maternal and neonatal outcomes	187	Intubation	114 intubated 73 non intubated	76 obese 35/76 intubated 41/76 non intubated	Not reported	Not reported	Obesity and need for intubation: cause-specific hazard ratio (CSH) 2.00, 95% CI (1.05–3.80), *p* = 0.03	Obesity associated with higher risk of intubation
8	Peter 2022 [53]	USA	To investigate the impact of maternal characteristics upon COVID-19 outcome, as well as whether disease severity impacted pregnancy outcomes	34	Symptomatic: fever, cough, myalgia, anosmia, congestion, headache, chills, dyspnea, nausea, vomiting, malaise	19 symptomatic	5 obese, all symptomatic	Not reported	Not reported	BMI of symptomatic vs. asymptomatic: 35.71 vs. 26.79, *P* = 0.004;	High BMI is associated with symptomatic COVID-19
9	Prasannan 2021 [54]	USA	To determine social determinants of health associated with severe acute respiratory syndrome due to COVID-19	544	Mild disease: no shortness of breath, dyspnea, or abnormal chest imaging Moderate disease: lower respiratory disease and oxygen saturation of ≥94% on room air Severe disease: oxygen saturation <94% on room air, ratio of arterial oxygen partial pressure to fraction of inspired oxygen < 300 mm Hg, respiratory frequency >30 breaths/min, or lung infiltrates >50% Critical disease: respiratory failure, septic shock, or MOF	115/544 with mild or moderate disease 70/544 with severe disease	283/544 obese	Not reported	Not reported	Mean BMI: 32.7 (women with severe to critical) vs. 30.9 (asymptomatic; *P* < 0.04	BMI associated with disease severity
10	Sakowicz 2020 [55]	USA	To compare clinical characteristics of pregnant women with and without severe acute COVID-19 disease	101	Symptomatic: fever, shortness of breath, cough, sore throat, body aches, chills, vomiting, diarrhea, loss of taste or smell, red or painful eyes	77/101 symptomatic	35/101 obese 19/35 symptomatic	Not reported	Not reported	Obesity and COVID-19 positivity *P* = 0.002	Women positive for COVID-19 were more likely to be obese
										Obesity and severity of symptoms *P* = 0.95	No significant differences between women with and without symptoms as regards BMI and obesity
11	Savasi 2020 [56]	Italy	To investigate the clinical evolution of COVID-19 disease in hospitalized pregnant women and factors associated with severe maternal outcome	77	Severe: urgent delivery based on maternal respiratory function or ICU or sub intensive care admission or both	14/77 with severe disease; 6/77 admitted to ICU	7/14 with severe disease were obese	Not reported	Not reported	BMI non severe vs. severe: 30 (19.4–54.1) vs. 22.8 (17.5–54.1) *p* = 0.02	High BMI associated with severe disease
12	Souza 2022 [57]	Brazil	To evaluate the effect of COVID-19 infection on obstetrical outcomes	289	SARS (severe acute respiratory syndrome)	47 SARS 241 no SARS	68 overweight (10/68 SARS) 72 obese (16/72 SARS)	Not reported	Not reported	RR of SARS 4.34 (1.04–19.01) for overweight, 6.55 (1.57–27.37) for obesity	Being overweight or obese is associated with higher risk of SARS
13	Torres-Torres 2022 [26]	Mexico	To evaluate the association of comorbidities and socioeconomic determinants with COVID-19-related mortality and severe disease in pregnant women in Mexico	13,062	**Severe pneumonia:** American Thoracic Society criteria **ICU admission** **Intubation**	176 deaths due to COVID-19 322 were admitted to ICU 1191 were diagnosed with severe pneumonia 185 were intubated	1016 obese; 30/176 deaths were obese	**Reported**	Not reported	Pneumonia RR 1.35 (1.14–1.59), *p* < 0.001	Obesity is a risk factor for severe COVID-19 pneumonia
										Intubation RR 1.37 (0.92–2.04) *P* = 0.122	Obesity is not a risk factor for intubation
										Severity of COVID-19 and BMI *P* = 0.17	BMI is not associated with severity of COVID-19 disease
14	Vimercati 2022 [36]	Italy	To evaluate the maternal and perinatal outcomes of COVID-19 infection during pregnancy	122	Mild symptoms: requiring non-invasive respiratory support Severe symptoms: requiring ICU admission	47 symptomatic 23/47 with mild to severe symptoms	69 overweight, 25 obese	Not reported		Symptoms: OR 1.66 (1.19–2.31) *p* < 0.001 for overweight OR 1.72 (1.22–2.41) *p* < 0.001 for obese	Symptomatic women more frequent among overweight or obese
15	Vousden 2021 [58]	UK	To compare incidence, characteristics, and outcomes of hospitalized pregnant women with symptomatic and asymptomatic COVID-19 vs. pregnant women without COVID-19	1148	Symptomatic: any among fever, cough, sore throat, breathlessness, headache, fatigue, limb or joint pain, vomit, rhinorrhea,, diarrhea, anosmia, pneumonia	722 symptomatic 63/722 required critical care 8/722 died	237 overweight 235 obese	Not reported	Not reported	Symptoms: OR 1.66 (1.19–2.31) *p* < 0.001 for overweight OR 1.72 (1.22–2.41) *p* < 0.001 for obese	Symptomatic women more frequent among overweight or obese

**Table 2:** Related maternal mortality.

	First Author	Country	Objective(s)	Women with COVID-19	Definition of Disease Severity	Disease Severity Status	Obese/Overweight Women	Mortality in Obese Women	Obstetric Outcome in Obese Women	Findings	
										Results	Comments as Reported in the Paper
1	Galang 2021 [45]	USA	To determine risk factors for illness severity among pregnant women with COVID-19	7950	Critical: mechanical ventilation/intubation, ECMO, ICU admission, ARDS, respiratory failure, septic shock, MOF, COVID-19 listed as cause of death Moderate-to-severe: not meeting criteria for critical; presence of dyspnea/shortness of breath and at least one among fever or cough Mild: symptomatic not meeting criteria of critical or moderate-to-severe	512 with moderate-to severe or critical illness	1974 obese		Not reported	Moderate to severe or critical disease and death RR 1.36 (1.23–1.51)	Pre-pregnancy obesity associated with moderate-to-severe or critical COVID-19 disease
2	Leal 2021 [31]	Brazil	To analyze maternal morbidity and mortality due to severe acute respiratory infections, including COVID-19	5469	Not reported	362/5469 died	264 obese: 44/264 died	**16.6%**	Not reported	Obesity among women who died 12.1% vs. 4.4%	In women with COVID-19, obesity was more common among those who died than among survivors
3	Takemoto 2020 [34]	Brazil	To describe clinical characteristics of pregnant women with severe COVID-19 and to examine risk factors for mortality	978	Not reported	978 symptomatic	43 obese		Not reported	OR = 2.31; 95% (CI 1.10–4.84) for obesity as a risk factor for maternal death	Obesity was one of the main risk factors for maternal death by COVID-19

**Table 3:** Association of overweight/obesity with COVID-19 related hospitalization and Internsive Care Unit (ICU) admission.

	First Author	Country	Objective(s)	Women with COVID-19	Definition of Disease Severity	Disease Severity Status	Obese/Overweight Women	Mortality in Obese Women	Obstetric Outcome in Obese Women	Findings	
										Results	Comments as Reported in the Paper
1	Budhram 2021 [59]	South Africa	To describe the risk factors and outcomes of pregnant women infected with COVID-19	673	Hospitalized for COVID-19	217 admitted to hospital for COVID-19 106 requiring critical medical care; 32 deaths	108 overweight 253 obese	14.7%	Not reported	BMI and hospital admission *P* = 0.16	BMI is not a risk factor for admission to hospital due to COVID-19
2	Doyle 2022 [23]	USA	To estimate the risk of COVID-19 infection in pregnancy and adverse maternal and perinatal outcomes	12,976	Need for ICU	Need for ICU 48/12,976	3455 overweight, 2079 Class I obesity 1048 Class II obesity 762 Class III obesity 12/14 maternal deaths involved obese patients		Not reported	Obesity and adverse composite outcome: OR 2.124 (1.381–3.268) *p* = 0.0006	Obesity associated with increased risk of adverse composite outcome
										Obesity and ICU admission: OR 1.910 (1.227–2.974), *p* = 0.0041	Obesity associated with increased risk of ICU admission
3	Mendez-Dominguez 2021 [24]	Mexico	To analyze the clinical course of pregnant women hospitalized for COVID-19 disease	42,525	Pneumonia Need for ICU	7064 hospitalized 1586 pneumonia 254 needed ICU 197/7064 died	637 obese 32/637 died	5%	Not reported	Admission to ICU and obesity OR 1.17 (0.75–1.81) *p* = 0.01	Obese COVID-19 patients -choose were significantly more prone/likely -choose to be admitted to the ICU
										ICU admission: Overweight, 1.15 (.87–1.59) Obesity class 1, 1.16 (.79–1.70) Obesity class 2, 1.27 (.79–2.04) Obesity Class 3, 2.30 (1.49–3.55)	Risk of ICU admission increased with increasing levels of pre-pregnancy obesity
4	Menezes 2020 [50]	Brazil	To evaluate clinical and social risk factors associated with negative outcomes of COVID-19 disease in pregnancy	2475	Adverse composite outcome: critical disease leading to death or admission to ICU or mechanical ventilation	590 adverse outcome	116 obese; 48/116 with adverse composite outcomes	Included in adverse composite outcome	Not reported	Obesity and need for intubation: cause-specific hazard ratio (CSH) 2.00, 95% CI (1.05–3.80), *p* = 0.03	Obesity associated with higher risk of intubation
5	Péju 2022 [52]	France	To assess the ventilatory management of pregnant women with COVID-19 admitted to the ICU and report on maternal and neonatal outcomes	187	Intubation	114 intubated 73 non intubated	76 obese 35/76 intubated 41/76 non intubated	Not reported	Not reported	ICU admission RR 1.17 (0.85–1.61), *p* = 0.321	Obesity is not a risk factor for ICU admission
6	Torres-Torres 2022 [26]	Mexico	To evaluate the association of comorbidities and socioeconomic determinants with COVID-19-related mortality and severe disease in pregnant women in Mexico	13,062	Severe pneumonia: American Thoracic Society criteria ICU admission Intubation	176 deaths due to COVID-19 322 were admitted to ICU 1191 were diagnosed with severe pneumonia 185 were intubated	1016 obese; 30/176 deaths were obese	Reported	Not reported	Intubation RR 1.37 (0.92–2.04) *P* = 0.122	Obesity is not a risk factor for intubation
										ICU admission RR 1.17 (0.85–1.61), *p* = 0.321	Obesity is not a risk factor for ICU admission

**Table 4:** Association of overweight/obesity with pregnancy outcomes in Covid-19 patients.

	First Author	Country	Objective(s)	Women with COVID-19	Definition of Disease Severity	Disease Severity Status	Obese/Overweight Women	Mortality in Obese Women	Obstetric Outcome in Obese Women	Findings	
										Results	Comments as Reported in the Paper
1	Stenton 2022 [37]	UK	To assess pregnancy outcomes of patients with COVID-19 placentitis	59 mothers, 61 newborns 47/59 positive at the time of labor	Placenta with positive immunohistochemical staining for COVID-19 spike protein in the syncytiotrophoblast	59/59 with placentitis	15/59 obese	Not reported	Pregnancy loss (miscarriage or stillbirth)	Pregnancy loss 67% (10/15) in obese versus 41% (14/34) In non-obese	Obesity associated with pregnancy loss
2	Vimercati 2022 [36]	Italy	To evaluate the maternal and perinatal outcomes of COVID-19 infection during pregnancy	122	Mild symptoms: requiring non-invasive respiratory support Severe symptoms: requiring ICU admission	47 symptomatic 23/47 with mild to severe symptoms	69 overweight, 25 obese	Not reported		Preterm birth and BMI *P* = 0.03	High BMI associated with preterm birth

**Table 5:** Association of micronutrient levels/supplementation with COVID-19 illness severity.

	First Author	Country	Objective	Population	Results	Details	Additional Comments
1	Anuk 2020 [39]	Turkey	To evaluate the status of zinc, copper and magnesium in pregnant women diagnosed with COVID-19 infection	100 COVID-19 positive 100 COVID-19 negative	*p*: 0.018	Disease severity correlation with zinc/copper ratio in COVID-19 +	In the first and third trimesters serum zinc levels were lower, serum copper levels were higher, the Zn / Cu ratio decreased and serum magnesium levels were higher in the COVID-19 positive group In the second trimester COVID-19 patients had lower serum zinc and copper levels compared to negative controls Zn/Cu ratio showed correlation with inflammatory and acute phase markers including IL-6, CRP, ESR, procalcitonin
					*p* < 0.0001	Serum Magnesium level significantly higher in COVID-19 +	
					*p*: 0.004	Serum zinc levels significantly lower in COVID-19 +	
					*p*: 0.006	Serum copper levels higher in COVID-19 +	
					*p*: 0.0004	In the second trimester copper levels decreased in COVID-19 +	
					*p*: 0.05	In the second trimester serum zinc levels were lower in COVID-19 +	
					*p*: 0.07	Disease severity correlated with serum zinc levels	
3	Bahat 2020 [40]	Turkey	To measure serum Vit D, Vit B12, and zinc levels in COVID-19 positive pregnant women	44 COVID-19 positive women	Mean serum Vit D, zinc, and Vit B12 levels *p* < 0.01	Mean serum levels of Vit D, zinc and Vit B12 were significantly lower than the accepted cut-off values	Patients with low serum levels of Vit D, zinc and Vit B12 may be more susceptible to COVID-19 infection
4	Erol 2021 [43]	Turkey	To evaluate the maternal serum afamin and vitamin E levels in pregnant women with COVID-19 and to investigate their association with composite adverse perinatal outcomes	60 COVID-19 positive 36 COVID-19 negative	*p* < 0.001, *p* < 0.001, and *p* = 0.004, respectively	Vitamin E levels were lower in COVID-19 + in all trimesters	Afamin levels were higher and vitamin E levels were lower in COVID-19 + pregnant women. This may support elevated oxidative stress and be related to composite adverse perinatal outcomes
					*p* > 0.05	Afamin levels were higher in COVID-19 + in all trimesters without reaching statistical significance	
					r = 0.264	Positive significant correlation between afamin and C-reactive Protein levels	
5	Erol 2021 [44]	Turkey	To assess the selenium status of pregnant women with COVID-19 and the effects of potential deficiency in serum selenium levels	71 COVID-19 positive 70 COVID-19 negative	*P* = 0.0003 and *P* = 0.001, respectively	Serum selenium levels of pregnant women in the second and third trimesters were lower in COVID-19 +	Serum selenium levels gradually decreased during the pregnancy; this decrease was enhanced in COVID-19 + patients, possibly due to needs depending on the immune response against infection. The decrease in maternal selenium levels was related to IL-6 and D-dimer levels, which indicate selenium’s role in disease progression
					*P* = 0.0002 for correlation with D-dimer, *P* = 0.02 for correlation with IL-6	Maternal selenium levels negatively correlated with D-dimer and interleukin-6 (IL-6)	
					*P* = 0.03	In the third trimester, maternal selenium negatively correlated with C-reactive protein levels	
6	Tekin 2022 [33]	Turkey	To investigate the association between Vit D and the clinical severity of COVID-19 in pregnant women	147 COVID-19 positive 300 COVID-19 negative	RR = 0.568, 95% CI [0.311–1.036]; *p* = 0.065; After excluding patients on vitamin supplementation: RR = 0.625, 95% CI [0.275–1.419]; *p* = 0.261	The clinical severity of COVID-19 disease was not affected by Vit D deficiency	The clinical severity of COVID-19 does not appear to be associated with vitamin D status in pregnant women
					RR 0.767 (95% CI [0.570–1.030]; *p* = 0.078	Testing positive for COVID-19 was not related to Vit D status	
					RR = 0.954; 95% CI [0.863–1.055]: *p* = 0.357	Pulmonary involvement of COVID-19 was similar between patients with Vit D deficiency and adequate Vit D levels	
					Vit D levels in COVID-19 + 10.35 [8.27] ng/mL vs. 19.02 [8.35] ng/mL in COVID-19 -; *p* < 0.05	Serum Vit D levels were significantly lower in COVID-19 + pregnant women	
7	Schmitt 2022 [45]	France	To evaluate the serum oxidative stress status of pregnant women with and without COVID-19, their inflammatory status, and their serum Vit D levels	15 COVID-19 positive (7 asymptomatic, 8 symptomatic) 20 COVID-19 negative	*p* > 0.05	No significant differences between asymptomatic COVID-19 + and COVID-19 -	Vit D deficiency during the third trimester of pregnancy was more marked in COVID-19 +
					*p* = 0.05	Significantly decreased Vit D levels in COVID-19+	
					*p* = 0.003	Low magnesium intake (<450 mg) was an independent risk factor for a weak immune response	
8	Citu 2022 [38]	Romania	To determine the effect of magnesium and magnesium-containing nutritional supplements on the immune response following COVID-19 infection in pregnant women, as well as to observe differences in pregnancy outcomes based on the supplements taken during pregnancy	448 COVID-19 positive 61/448 took magnesium-only supplements 74/448 took a combination of calcium, magnesium, and zinc 313/448 had no supplementation	*p* = 0.868	COVID-19 severity was similar in the three study groups	Pregnant women who supplemented their diet with calcium, zinc, and magnesium, or magnesium only did not have a different clinical course of COVID-19 disease, but no supplementation led to a weaker immune status
					14.4% vs. 6.6% vs. 5.4%, *p* = 0.038	Significantly higher proportion of premature births in the group of COVID-19 pregnant women who did not supplement their diet compared with those who took magnesium supplements	
					Zinc: 0.97 (95% CI: 0.87–1.08), *P* = 0.55 Copper: 1.07 (95% CI: 1.00–1.14), *P* = 0.06	Circulating zinc and copper levels show limited evidence of association with COVID-19 infection	
9	Sobczyk 2022 [41]	UK	To test whether genetically predicted Zn, Se, Cu or vitamin K1 levels have a causal effect on COVID-19-related outcomes, including risk of infection, hospitalization and critical illness		Hospitalization and: Vitamin K1: 0.98 (95% CI: 0.87–1.09), *p* = 0.66 Copper: 1.07 (95% CI: 0.88–1.29), *P* = 0.49 Critical Illness and: Vitamin K1: 0.93 (95% CI: 0.72–1.19), *p* = 0.55 Zinc: 1.21 (95% CI: 0.79–1.86), *P* = 0.39	Hospitalization and critical illness outcome are poorly related with circulating levels of vitamin K1, copper and zinc	No evidence that supplementation with zinc, copper or vitamin K1 can prevent COVID-19 infection, critical illness or hospitalization
					73/82 (89%) COVID-19 + had vitamin D deficiency vs. 131/174 (75.3%) in COVID-19-*P* = 0.01	Vitamin D deficiency is more frequent in COVID-19 + pregnant women	
10	Ferrer-Sanchez 2022 [42]	Spain	To establish a relationship between serum Vit D levels and COVID-19 in pregnant women	82 COVID-19 positive (75 mild symptoms, 7 moderate, severe or critical symptoms) 174 COVID-19 negative			Relationship between vitamin D deficiency in pregnant women and COVID-19 infection

## 4. Discussion

In 2021, the WHO stated that overweight (BMI > 25 kg/m^2^) and obesity (BMI > 30 kg/m^2^) are major risk factors for a relevant number of chronic diseases. These body weight abnormalities affect 40 and 15% of the general population, respectively, with a slightly higher prevalence among women. Nowadays, approximately 28% of pregnant women are overweight, and 11% are obese. Maternal obesity has emerged as a key risk factor for obstetric complications in pregnant women.

Overweight and obesity per se represent well-known risk factors for several adverse obstetric outcomes, both maternal (pre-eclampsia, gestational diabetes, postpartum hemorrhage, etc.) and fetal (preterm birth, large-for-gestational-age infants, intrauterine death, etc.), which together increase maternal, fetal, and neonatal mortality and morbidity. Many health programs aimed at preventing gestational diseases rely on both adequate weight loss and normal body weight in the preconception period, as well as appropriate weight gain during pregnancy.

A higher-than-normal BMI implies a series of complex immunologic, metabolic, and endocrine changes that also affect the immune response to viral infections [9]. It is reasonable to assume that there could be a relationship between overweight/obesity and susceptibility to COVID-19 infection, its severity, and its impact on pregnancy. Indeed, a review of the available literature suggests that there is a positive correlation between overweight/obesity and COVID-19 incidence and severity during pregnancy. Although none of the published studies were specifically designed to detect such an association, it was clearly evident after extrapolating data of pregnant women from large, published databases.

Pregnancy is a key moment for both physical and neurocognitive fetal growth; any nutritional imbalance or deficiency of important nutrients could lead to insufficient and/or impaired fetal development, thus, increasing the risk of unfavorable maternal, fetal, and neonatal outcomes. Micronutrients, such as vitamins A, C, D, and E, and minerals (Fe, Se, and Zn) can actively and effectively boost the immune system, thereby potentially preventing pregnancy complications. In particular, vitamin A is crucial for immune system development. Some authors demonstrated that for a few viral diseases, the supplementation of vitamin A led to a better prognosis and improved outcomes, including clearance of HPV lesions or a reduction in some measles-related complications [60]. Vitamin C protects against infections, vitamin D exerts anti-inflammatory and immunomodulatory effects, and vitamin E is mainly a strong antioxidant and immunomodulatory vitamin that decreases oxidative stress. Serum levels of micronutrients progressively decrease with gestational age due to both physiological hemodilution and increased maternal–fetal demand. Although the available data concerning micronutrients in pregnant women infected with COVID-19 are very heterogeneous and have been obtained in small groups of patients, they suggest the possible existence of a relationship between micronutrient deficiency and the severity of COVID-19 disease, as well a potential role for micronutrient supplementation in preventing and/or attenuating the impact of COVID-19 during pregnancy.

Meta-analysis suggests a net increase in risk (twice or more) for severe disease, maternal death for COVID-19, and hospital admission for COVID-19-related causes for obese pregnant women. Although the results are statistically significant, even if based on a relatively low number of studies, the causal relationship between obesity and adverse outcomes in COVID-19-positive pregnant women must be discussed.

When considering the dose–response criterion, data are in favor of a causal relationship, as with the increase in the obesity class, the risk of adverse events increases too.

There is also a biological plausibility for the association between obesity and adverse COVID-19 outcomes, as we note the same association in different populations, e.g., the general population. In a review on obesity and COVID-19, obesity emerges as one of the major risk factors for COVID-19 severity. According to the review, adiposity-related systemic inflammation, involving cytokine, chemokine, leptin, and growth hormone signaling, and the involvement of hyperactivation of the renin–angiotensin–aldosterone system (RAAS) could play a key role [61].

On the other hand, poor pregnancy outcomes (pre-eclampsia, preterm birth, and stillbirth) are more frequent in COVID-19-positive pregnant women [3]. Furthermore, it is already well known that maternal and pregnancy outcomes are negatively influenced by obesity by increasing maternal and fetal/neonatal morbidity and mortality [62,63,64]. We suppose that obesity could act as a further risk factor in COVID-19-positive pregnancies; unfortunately, we could not perform stratified analysis and meta-regression in order to isolate the impact of obesity, which, in this case, is probably overestimated.

COVID-19 is presently still a worldwide emergency, with thousands of new cases every day, and unfortunately, it may not be the only pandemic we will face, so greater knowledge regarding how to deal with a similar situation could be useful in the future. With these assumptions, we can, therefore, hypothesize the application of a Research Agenda, by creating a list of data to be collected in order to conduct research in which both maternal and fetal outcomes have to be reported, underlying the importance of considering the mother–fetus dyad as a single entity with specific needs.

We suggest that at least the following parameters be considered:

Baseline maternal data: age, ethnicity, BMI, parity, and pre-existing diseases;

Data during pregnancy: gestational age at COVID-19 (or other) infection, symptoms of infection, hospitalization, major complications, maternal death, pre-eclampsia, any other pregnancy complications, and maternal ICU admission;

Delivery data: gestational age at delivery, the reason for induction or for cesarean section, and blood loss at delivery;

Neonatal data: neonatal birthweight, birthweight centile, APGAR score, malformations, stillbirth/neonatal death, admission to neonatal intensive care unit, duration of hospitalization, and other neonatal complications.

Such a common core of data would be useful for carrying out comparisons across settings, as well as for testing new hypotheses and new approaches.

### Limitations

We did not register our study with the International Prospective Register of Systematic Reviews (PROSPERO).

As we have highlighted, our study has limitations, some of which are inherent to the original studies. These limitations are likely due to the lack of time during the pandemic and the need to rapidly share experiences. Furthermore, some studies did not involve gynecologists or obstetricians, and thus, pregnancy and fetal outcomes and, as a consequence, pregnancy and/or neonatal complications were underreported.

As the data were observational, we could not control for possible residual confounding.

## 5. Conclusions

COVID-19 infection can influence the outcome of pregnancy both for the mother and the fetus: its effects may be amplified in women with an impaired nutritional state, including overweight/obesity or by deficiency of macro- and micronutrients.

Further data are needed to better understand how body weight and nutrition can influence the prognosis of COVID-19 in pregnancy, and how pre-pregnancy normalization of BMI and tailored nutritional supplementation can affect pregnancy outcomes [24,26,34,35,36,37,46,47,48,49,50,51,52,53,54,55,56,57,58,59].

## Figures and Tables

**Figure A1:**
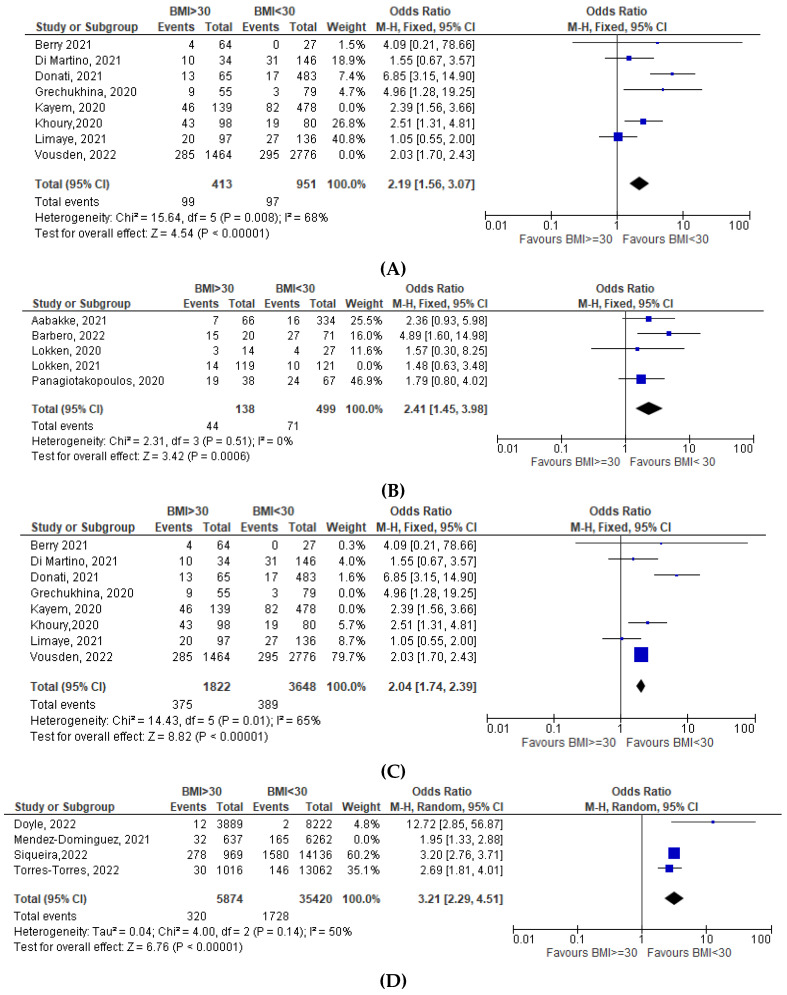

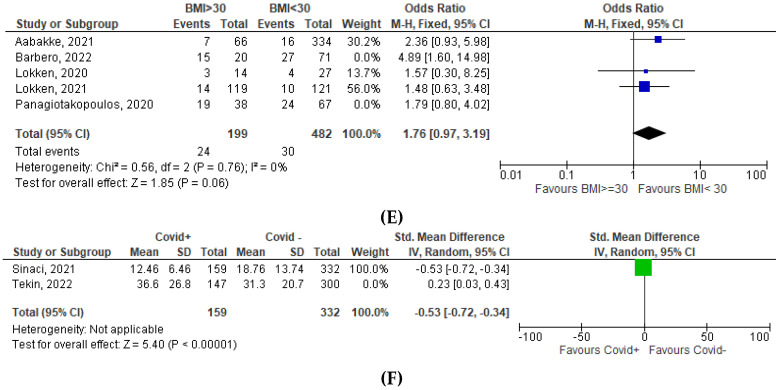
(**A**) Sensitivity analysis including small sample size studies (<100 per arm) and using a fixed effect model. BMI >= 30 vs. BMI <3 0—Disease severity [15,16,17,18,19,20,21,22]. (**B**) Sensitivity analysis including small sample size studies (<100 per arm) and using a fixed effect model. BMI >= 30 vs. BMI < 30—Hospital admissions [27,28,29,30,31]. (**C**) Sensitivity analysis including only good quality studies—Disease severity. BMI >= 30 vs. BMI < 30 –. [15,16,17,18,19,20,21,22]. (**D**) Sensitivity analysis including only good quality studies—Maternal death. [23,24,25,26]. (**E**) Sensitivity analysis including only good quality studies—Hospital admission. [27,28,29,30,31]. (**F**) Sensitivity analysis including only good quality studies—Vit D serum levels [32,33].

**Figure A2:**
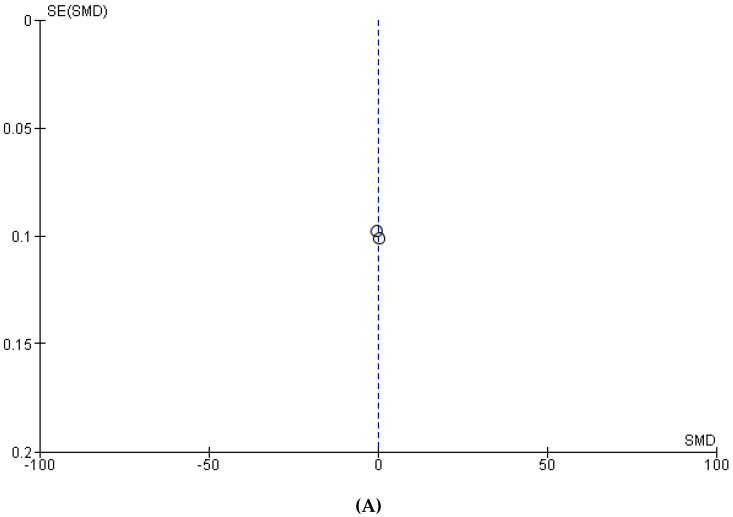

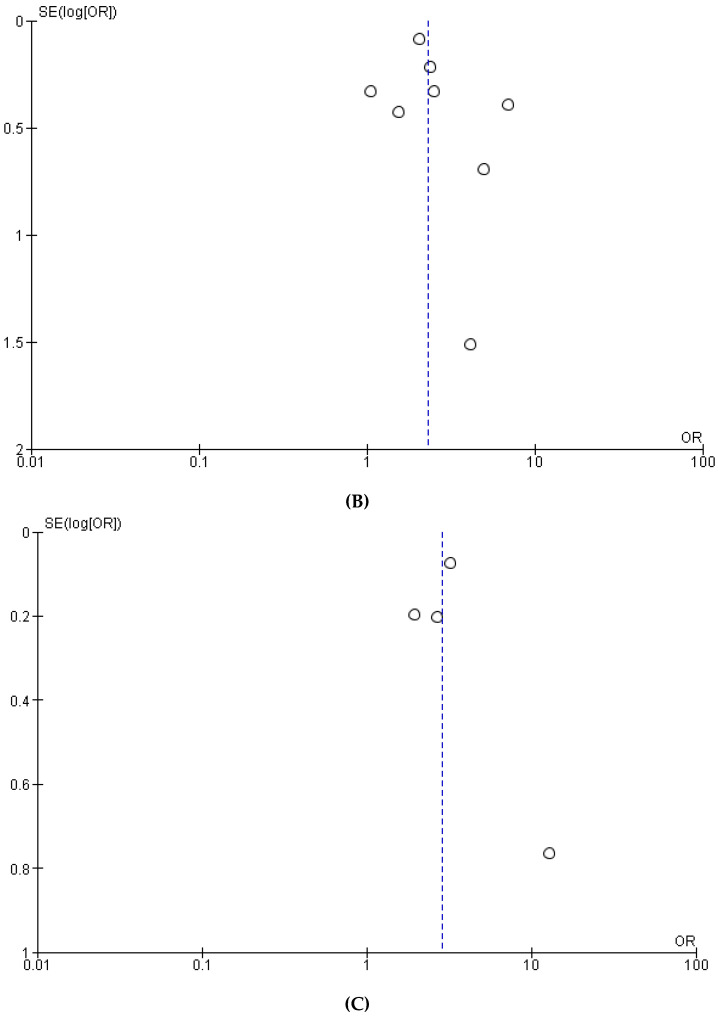

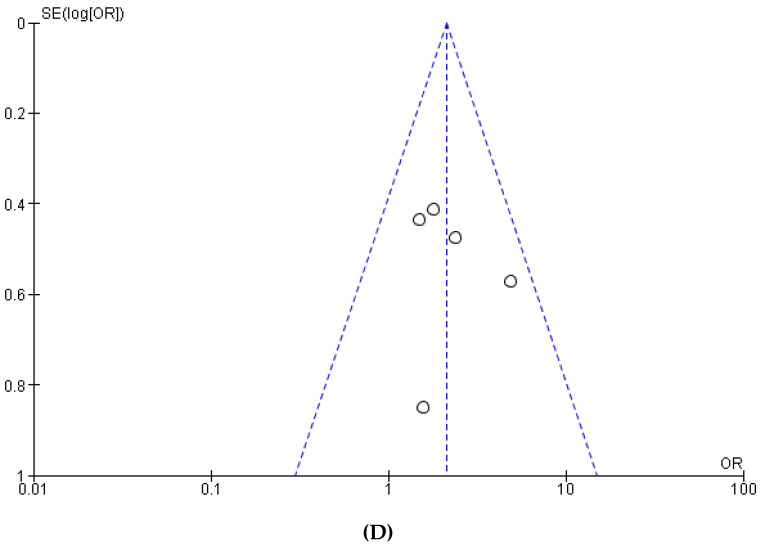
(**A**) Covid+ pregnant women vs. Covid- pregnant women, outcome: 1.1 Vitamin D serum levels. Funnel plot. (**B**) Funnel plot of comparison: 2 BMI >= 30 vs. BMI < 30, outcome: Disease severity. (**C**) Funnel plot of comparison: 2 BMI >= 30 vs. BMI < 30, outcome: Maternal death. (**D**) Funnel plot of comparison: 2 BMI >= 30 vs. BMI < 30, outcome: Hospital admission.
